# The Interaction Effects of Aeration and Plant on the Purification Performance of Horizontal Subsurface Flow Constructed Wetland

**DOI:** 10.3390/ijerph19031583

**Published:** 2022-01-30

**Authors:** Xinyi Chen, Fei Zhong, Yue Chen, Juan Wu, Shuiping Cheng

**Affiliations:** 1 Key Laboratory of Yangtze River Water Environment, Ministry of Education, College of Environmental Science and Engineering, Tongji University, 1239 Siping Road, Shanghai 200092, China; chenxinyi0524@163.com (X.C.); wujuan789@tongji.edu.cn (J.W.); 2 School of Life Science, Nantong University, 9 Seyuan Road, Nantong 226019, China; 3 Hebei Construction Group Installation Engineering Co., Ltd., Baoding 071051, China; chenyuehbjs@126.com; 4 Institute of Eco-Environmental Engineering, Tongji University, 1239 Siping Road, Shanghai 200092, China

**Keywords:** constructed wetland, domestic sewage, aeration position, plant species

## Abstract

Aeration and plants exhibit influence on the water purification performance in constructed wetlands (CWs). However, the interaction between aeration and plants on enhancing performance of domestic sewage treatment is unclear. Our study aims to optimize the combination of aeration position and plant species, promoting the extensive and effective application of CWs. Herein, six horizontal subsurface flow (HSSF) CWs small scale plots were established and divided into two groups according to the plant (i.e., *Canna indica* and *Iris sibirica*). To adjust the distribution of dissolved oxygen (DO) in CWs, each group had three plots of HSSF CWs. One plot was aerated at the bottom of the first quarter of the filtration chamber, one plot was aerated at the bottom of the inflow chamber, and the remaining plot was not aerated as a control. Results showed that aeration at the bottom of the first quarter filtration chamber could contribute to the highest removal efficiency of chemical oxygen demand (COD), ammonium nitrogen (NH_4_^+^-N) and total nitrogen (TN). The COD, NH_4_^+^-N, and TN removal percentages decreased with the drop in temperature. However, the plot aerated at the bottom of the first quarter filtration chamber with *I. sibirica* exhibited the best average COD_Cr_, NH_4_^+^-N and TN removal percentages in both the warm season (83.6%, 82.7% and 76.8%) and the cool season (66.3%, 44.1% and 43.8%). Therefore, this study indicated that the combination of aerating at the bottom of the first quarter filtration chamber and planting with *I. sibirica* in the HSSF CWs would be a promising way forward for wastewater treatment, especially in low temperature seasons.

## 1. Introduction

The carbon, nitrogen and phosphorus concentrations in the water environment are mainly affected by the anthropogenic emissions [1,2], which are responsible for the eutrophication of water bodies. To alleviate the water environment crisis, great attention has been placed into the development of novel ways to improve water quality.

Constructed wetlands (CWs) are complex ecosystems composed of plants, microorganisms and substrates to remove contaminants [3]. CWs have the advantages of low investment cost, convenient operation and management, high ecological benefits, good pollutant removal effects, and wide applications [4]. Therefore, CWs have been gradually applied to domestic sewage, mine acid drainage and agricultural runoff treatment since the 1960s. CWs can be classified as surface flow and subsurface flow systems, which are depended on water levels. Furthermore, subsurface flow CWs are divided into two types: vertical subsurface flow (VSSF) and horizontal subsurface flow (HSSF) systems [5]. The pollutant types, contaminant concentrations, geographical environment etc. should be taken when selecting the type of CWs.

Physical, chemical, and biological processes occur simultaneously in HSSF CWs to remove contaminants [6]. Biological process plays an important role in removing organic matters and nitrogen. Organic matter is largely removed by microbial degradation, while nitrogen is mainly removed by ammonization, nitrification and denitrification in different areas of HSSF CWs with suitable redox conditions. Subsurface oxygen limitation has been identified as one of the main factors compromising contaminant removal in HSSF CWs [7].

Plants play an important role in the process of purification in CWs. The main way of natural reoxygenation in CWs is oxygen secretion from plant roots, and this ability is greatly related to plant species [8]. Plants will also affect the microbial community structure, activity and spatial distribution in the rhizosphere in CWs, thus affecting the water purification performance [4,9]. Previous studies reported that the ammonium oxidation and microbial respiration rates in the rhizosphere was related to the oxygen secretion by plant roots [10]. Results indicated that the higher levels of dissolved oxygen (DO), the higher abundances of the genes related to biodegradation that were found in the rhizosphere [10]. In this research, we selected two emergent plant species (*Iris sibirica* and *Canna indica*) to investigate whether plant species affect the DO value in the plots and what the different purification performances are between the two species.

Oxygen secreted from the plant root is generally not sufficient enough to support the removal of contaminants such as ammonium nitrogen (NH_4_^+^-N) [11]. Artificial aeration is considered to be the most effective way to improve the redox conditions in CWs. The effects of the aeration site [12,13], aeration intensity [14,15], aeration pattern [16,17] and plant species [18,19] on the treatment efficiencies of HSSF CWs were reported individually. In terms of aeration position, there are generally three preferable sites, i.e., at the front, middle and rear section of HSSF CWs. A previous study has shown that front aeration greatly improved organic and nitrogen (N) removal compared with middle and rear aeration [12]. However, the potential interaction effects of aeration position and plant species on the performance of HSSF CWs have not been evaluated. The objective of this study is to assess the individual and combined effects of aeration position and plant species on the purification performance of HSSF CWs.

## 2. Materials and Methods

### 2.1. Experimental Setup

**Figure 1:**
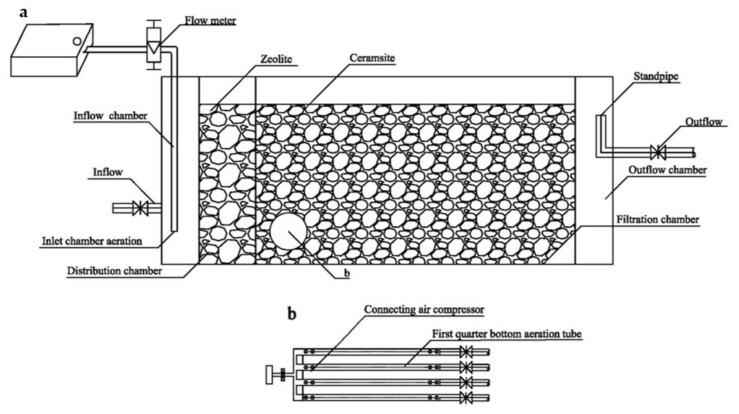
Schematic of the horizontal subsurface flow constructed wetland (HSSF-CW) plots. (**a**) Plots with aeration at the bottom of the inflow chamber. (**b**) the top view structure of the plots with aeration at the bottom of the first quarter filtration chamber.

Six small-scale horizontal subsurface flow constructed wetland (HSSF-CW) plots were constructed at the University of Tongji, Shanghai, China. Each plot (Figure 1) consisted of four chambers made of polyvinylchloride (120 × 40 × 60 cm). Along the water flow, it was separated into four parts: the water inflow chamber (10 cm), the distribution chamber (15 cm), the filtration chamber (85 cm) and the outflow chamber (7 cm). The filtering substrate was filled to 55 cm height. Zeolite with particle size of 8–12 mm was filled in the distribution chamber, and ceramsite with particle size of 6–8 mm was filled in the filtration chamber. The effluent water level was maintained by the standpipe, which was about 5 cm below the substrate surface. In order to verify interaction between plant and aeration, six HSSF-CW plots were divided into two groups according to plant species. A group of three plots were planted with *Canna indica*. Among them, one plot was aerated at the bottom of the first quarter filtration chamber (C3), one plot was aerated at the bottom of the inflow chamber (C2), and the remaining plot was not aerated as a control (C1). The second group of three plots were planted with *Iris sibirica*, which were divided into I3, I2 and I1, with the same settings as mentioned in the *C. indica* group.

The installation method of the aeration tubes in the plot at the bottom of the first quarter filtration chamber was shown in Figure 1b. Four aeration tubes (inner diameter 25 mm) were set at the bottom of the first quarter of the filtration chamber and evenly distributed 10 cm above the bottom of the system. All of the aeration tubes were connected to an air compressor (OUTSTANDING, 550 W- 8 L, Outstanding Industry and Trade Co., Ltd., Taizhou, China) through different branches of latex tubing. A row of bores (1 mm diameter) was evenly arranged in the upper side in the aeration tube wall. The schematic diagram of aerating at the bottom of the inflow chamber was provided in Figure 1a. Aeration was performed in the inflow chamber using a low-power air pump (BOYU, s-2000, Boyu Group Co., Ltd., Chaozhou, China). Flow meters were used to ensure that the aeration volume of each system was the same.

### 2.2. Operation Conditions

The concentrations of chemical oxygen demand (COD_Cr_), ammonia nitrogen (NH_4_^+^-N), total nitrogen (TN) and total phosphorus (TP) in the synthetic wastewater, which were prepared using CH_3_-COONa, CH_4_N_2_O, NH_4_Cl, KH_2_PO_4_ (analytical grade), were similar to the domestic sewage in China. The main parameters of the water quality were dissolved oxygen (DO): 0.13 ± 0.05 mg/L, oxidation-reduction potential (ORP): −181 ± 25.3 mV, COD_Cr_: 272 ± 45.7 mg/L, NH_4_^+^-N: 34.1 ± 3.98 mg/L, nitrite (NO_2_^−^-N): 0.09 ± 0.07 mg/L, and nitrate (NO_3_^−^-N): 0.35 ± 0.39 mg/L, TN: 49.8 ± 11.8 mg/L, TP: 2.93 ± 0.42 mg/L, respectively.

A feed tank with a 500 L capacity stored the synthetic wastewater. Peristaltic pumps (BT300–2J, Longer Precision Pump Co., Ltd., Baoding, China) were used to pump the wastewater to CWs to achieve a hydraulic loading rate of 100 mm/d. Intermittent aeration was used in both aeration positions, and the rate was 0.5 L/min. The experiment lasted from September 2020 to January 2021. To acquire information about the possible influence of seasonality on the performance of the system, the removal efficiencies were divided into two stages: temperature above 15 °C represented the warm season (S1) and temperature below 15 °C represented the cool season (S2).

### 2.3. Water Sampling and Analysis

To obtain a stable performance, all six plots were operated for three months before the experiment. Water samples were collected weekly from feed tank and effluents between 8:00 a.m. and 9:00 a.m. DO and ORP were measured in situ using a Thermo-Orion 5 Star portable meter (Thermo-Orion Inc., Waltham, MA, USA). Room temperatures were monitored in real time (JINGCHUANG temperature recorder, RC-5+, Jingchuang Co., Ltd. Shanghai, China). The samples were analyzed immediately in the laboratory for COD_Cr_, NH_4_^+^-N, NO_2_^−^-N, NO_3_^−^-N, TN and TP, followed the standard methods [20].

### 2.4. Statistical Analysis

All data were expressed as an average of three replicates with standard deviation. Water purification performance among the six plots was calculated by SPSS 26.0 (SPSS Inc., Chicago, IL, USA), including analysis of variance, homogeneity of variance and normality, and one-way ANOVA multiple comparisons for the mean removal percentages at 95% confidence level (*p* < 0.05). Two-way ANOVA was used to determine the individual and combined effects of aeration position and plant species on the purification performance of HSSF-CW plots.

## 3. Results and Discussion

### 3.1. DO, ORP and Temperature in the Six HSSF-CW Plots

**Figure 2:**
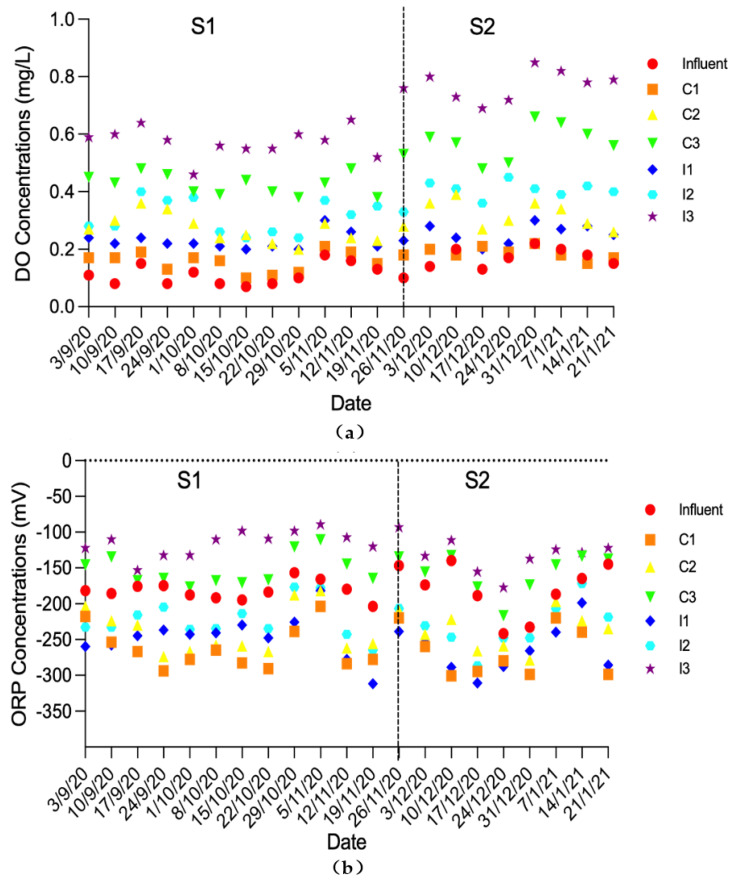

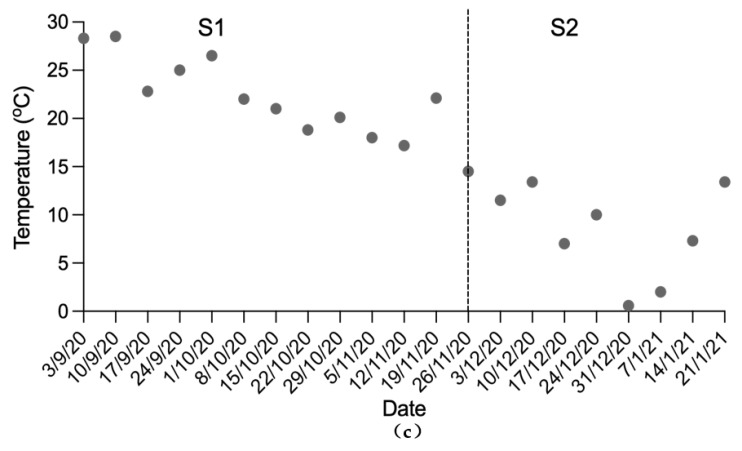
Variations of DO, ORP and temperature in the influent and effluent of the six plots. (**a**) DO; (**b**) ORP; (**c**) Temperature (T). The experiment was divided into two stages: S1 (T ≥ 15 °C) and S2 (T < 15 °C).

The physicochemical characteristics of the influent and effluent from the six HSSF-CW plots were summarized in Figure 2. The temperatures were above 15 °C before 26 November 2021; the stage (S1) from 3 September to 26 November represented the warm season. And the temperatures were below 15 °C after 26 November 2021, which was the initial of stage 2 (S2) to represent the cool season. The DO concentrations (0.13 ± 0.05 mg/L) and ORP values (−181 ± 25.3 mV) in the influents were relatively low, which had a negative effect on the nitrification process in CWs without optimization [21]. In this study, the aeration position and plant selection were considered for the optimization of the redox conditions in HSSF CWs.

As shown in Figure 2, the average DO concentrations in the effluents were in the order of C3 > C2 > C1 and I3 > I2 > I1. It indicated that aeration at the bottom of the first quarter filtration chamber plots (C3 and I3) could facilitate the reoxygenation of waterbodies and enhance the purification capacity of CWs [22]. In these two plots, oxygen was directly supplied in the filtration chamber, which can strengthen the synergistic effects of the substrate, plant and microorganism on the physical, chemical, and biological processes in the CWs [11,23,24]. However, the reoxygenation was poor in the non-aeration plots (C1 and I1), which were only re-oxygenated through the atmosphere and macrophytes. As a result, the DO concentrations in the non-aeration group (C1 and I1) were significantly lower than those in the plots aerated at the bottom of the first quarter filtration chamber (C3 and I3). The results also showed that *I. sibirica* had greater influence on the DO enhancement than *C. indica* did. In the plots aerated at the bottom of the first quarter filtration chamber, the average DO concentrations in the effluent were 0.49 mg/L and 0.66 mg/L for C3 and I3, respectively. Furthermore, the DO concentrations increased with the decrease of temperature at six HSSF-CW plots, which were similar to the results reported by Chen et al. [25]. Besides, DO concentrations in the plots aerated at the bottom of the first quarter filtration chamber were significantly affected by the temperature (C3, *p* < 0.05; I3, *p* < 0.05), while the effect of temperature on DO concentration in non-aeration group (C1 and I1) was relatively weak.

Due to the characteristics of domestic wastewater, the ORP in the influent was usually negative. In this study, passing through the HSSF CWs, the ORP values were in the order of C3 > C2 > C1 and I3 > I2 > I1, which followed the results of DO. It indicated that aeration was beneficial for the optimization of redox conditions in the HSSF CWs.

### 3.2. COD, N and P Removal from the Six HSSF-CW Plots

**Figure 3:**
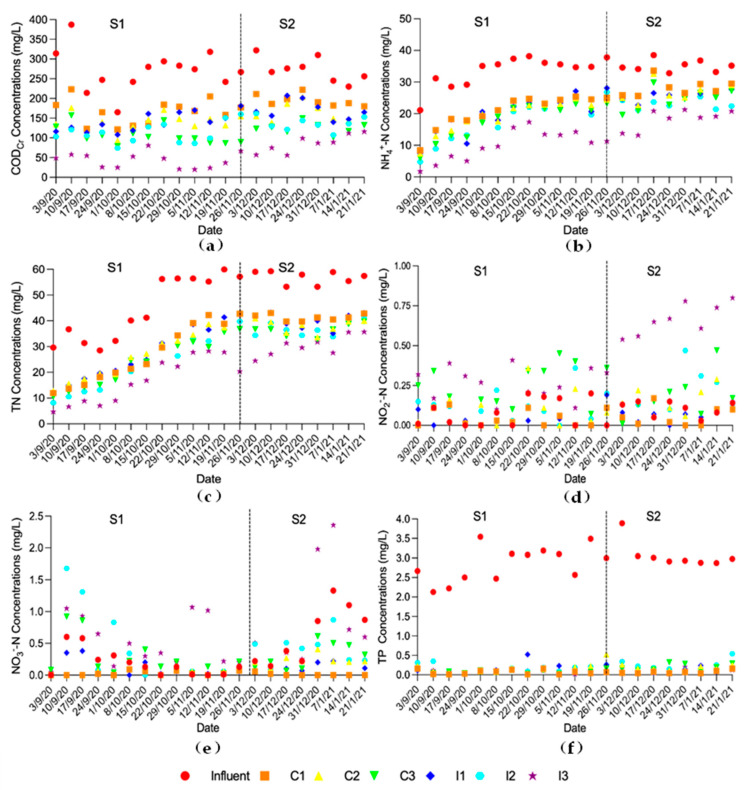
Variations of the water quality in the influent and effluent of the experiment plots. (**a**) COD_Cr_; (**b**) NH_4_^+^-N; (**c**) TN; (**d**) NO_2_^−^-N; (**e**) NO_3_^−^-N; (**f**) TP. The experiment was divided into two stages: S1 (T ≥ 15 °C) and S2 (T < 15 °C).

In this study, the lowest concentrations of COD_Cr_ (59.8 ± 29.4 mg/L), NH_4_^+^-N (13.3 ± 5.81 mg/L) and TN (22.0 ± 9.89 mg/L) in the effluent were observed in I3 (Figure 3), which was attributed to the combination of optimal aeration position and plant species.

The effluent NO_2_^−^-N and NO_3_^−^-N concentrations remained relatively low in the six HSSF-CW plots (Figure 3). The NO_2_^−^-N concentration in the effluent of the six HSSF-CW plots were relatively low, and were below 0.9 mg/L. The NO_3_^−^-N concentration in the effluent of the six HSSF-CW plots fluctuated greatly with the fluctuation of the influent NO_3_^−^-N concentration, but were less affected by temperature variation. The NO_3_^−^-N concentration in the effluent of the six HSSF-CW plots were below 2.36 mg/L in general. It indicated that neither NO_2_^−^-N nor NO_3_^−^-N accumulated in the six HSSF-CW plots. Therefore, the denitrification process was carried out efficiently in the six HSSF-CW plots.

HSSF CWs, filled with ceramsite, has been previously reported to be efficient at TP removal [26]. The concentrations of TP in the effluent were lower in C1 (0.07 ± 0.05 mg/L), C2 (0.16 ± 0.11 mg/L), C3 (0.12 ± 0.08 mg/L), I1 (0.15 ± 0.11 mg/L), I2 (0.19 ± 0.12 mg/L) and I3 (0.12 ± 0.07 mg/L), respectively. The concentrations of TP in the effluent met the Class III Standard in Surface Water Environmental Quality Standards (GB3838-2002, China) in the six HSSF-CW plots (TP < 0.2 mg/L).

### 3.3. Effects of Aeration Position and Plant Species on the Purification Performance on the Six HSSF-CW Plots

**Figure 4:**
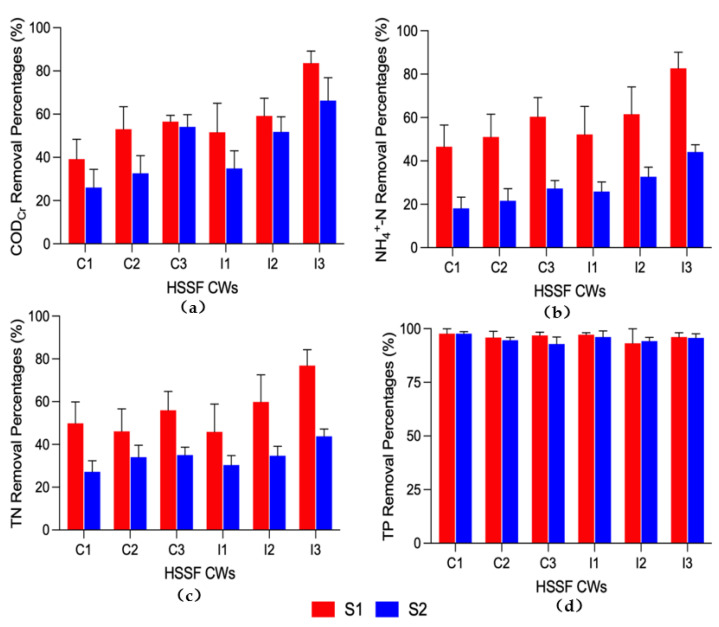
Total removal efficiencies of COD_C__r_, NH_4_^+^-N, TN and TP by HSSF-CW plots. (**a**) COD_Cr_; (**b**) NH_4_^+^-N; (**c**) TN; (**d**) TP. Vertical thin bars represent standard deviations (*n* = 5). The experiment was divided into two stages: S1 (T ≥ 15 °C) and S2 (T < 15 °C).

The effects of aeration position and plant species on the treatment efficiencies of the six HSSF-CW plots were shown in Figure 4. During the two stages, the COD_Cr_ removal percentages were in the order of C3 > C2 > C1 and I3 > I2 > I1. It indicated that although COD_Cr_ can be degraded both aerobically and anaerobically by micro-organisms in HSSF CWs [27], suitable aeration position significantly enhanced the COD_Cr_ removal efficiencies in the HSSF CWs planted with *I. sibirica* and *C. indica*, respectively. Wang et al. [28] also reported that suitable aeration position could be used to yield better results in reducing COD_Cr_ in wastewater. The average COD_Cr_ removal percentage in I1 (*I. sibirica*) was significantly higher than that in C1 (*C. indica*), which increased by 31.7% and 34.4% (S1 and S2 *p* < 0.05), respectively. The HSSF CWs planted with *I. sibirica* showed higher COD_Cr_ removal ability than that planted with *C. indica* did.

The COD_Cr_ removal percentages decreased with the temperature drop from S1 to S2 in the HSSF CWs (*p* < 0.05). But to compare the COD_Cr_ removal percentages of C1 (39.2%) in S1 with that of C3 (54.1%) in S2, the COD_Cr_ removal percentages even increased 38.0%. Similarly, comparing the COD_Cr_ removal percentages of I1 (51.6%) in S1 with that of I3 (66.3%) in S2, the COD_Cr_ removal percentages also increased 28.5%. That means aeration at the bottom of the first quarter filtration chamber could eliminate the negative effects at low temperature and improve the COD_Cr_ removal in HSSF CWs. As a result, the plot with the combination of aeration at the bottom of the first quarter filtration chamber and planting *I. sibirica* effectively enhanced COD_Cr_ removal efficiency under the two temperature seasons.

For the NH_4_^+^-N removal (Figure 4), the results showed that the removal percentages of NH_4_^+^-N were in the order of C3 > C2 > C1 and I3 > I2 > I1 during the two stages, following the trend of COD_Cr_ removal percentages. It was consistent with Zhang et al. [14], i.e., aeration can greatly enhance the removal efficiencies of HSSF CWs. The best NH_4_^+^-N removal performances in C3 and I3 mainly benefited from the highest DO concentrations in C3 and I3 (Figure 2), corroborating a recent report [11]. With the aeration at the bottom of the first quarter filtration chamber, the average NH_4_^+^-N removal percentages in I3 were significantly higher than those in C3, which increased by 37.0% and 61.5%, respectively (S1 and S2, *p* < 0.05). HSSF CWs planted with *I. sibirica* were more effective than those with *C. indica*. These results indicated that the plot with the combination of aeration at the bottom of the first quarter filtration chamber and planting *I. sibirica* had better NH_4_^+^-N removal efficiency.

In addition, the NH_4_^+^-N removal efficiencies sharply decreased as the temperature dropped (*p* < 0.05). Compared with those in S1, the NH_4_^+^-N removal efficiencies decreased 61.1% (C1), 57.6% (C2), 54.8% (C3), 50.3% (I1), 46.9% (I2) and 46.7% (I3) in S2, respectively. It might be attributed to the fact that the drop in temperature inhibited the activity of nitrifying bacteria [29]. But from the NH_4_^+^-N removal percentages of I1 (52.2%) in S1 and I3 (44.1%) in S2, it only decreased 15.5% in the cool season. It indicated that aeration at the bottom of the first quarter filtration chamber could alleviate the low temperature effects on NH_4_^+^-N removal in HSSF CWs.

The effects of aeration position on the TN removal efficiencies were consistent with that of the NH_4_^+^-N removal efficiencies during S1 stage, which were in the order of C3 > C2 > C1 and I3 > I2 > I1. The TN removal percentages of the six HSSF-CW plots were relatively low during S2 stage, which indicated the activity of denitrifying bacteria are greatly affected by environmental factors (e.g., temperature) [30]. Nevertheless, the TN removal percentages in I1 (30.4%), I2 (34.7%) and I3 (43.8%) (*I. sibirica* group) were better than those of C1 (27.2%), C2 (34.0%) and C3 (35.0%) (*C. indica* group), which were mainly due to the thriving of *I. sibirica*, and the withering of *C. indica* during S2 stage.

Ceramsite-filled HSSF CWs have been previously reported to be efficient on TP removal [25]. Bai et al. [31] and Dong et al. [32] reported that DO had no significant influence on TP removal. In this study, the average TP removal efficiencies in the six HSSF-CW plots filled with ceramsite were all above 90% (Figure 4). Herein, it indicated that neither aeration position nor plant species contributed to further enhancements in the TP removal efficiencies. The roles of aeration position or plant species on phosphorus removal might be covered by the adsorption capacity of ceramsite.

Two-way ANOVA showed that removal efficiencies of COD_Cr_, NH_4_^+^-N and TN in HSSF CWs were largely affected by both aeration position and plant species during the whole experimental period (Table 1., *p* < 0.05). The results of combined effect revealed that planting *I. sibirica* would further improve the COD_Cr_, NH_4_^+^-N and TN removal in HSSF CWs with the aeration at the bottom of the first quarter filtration chamber (77.5%, 62.1% and 57.6%). It showed a significant interaction term (i.e., aeration position × plant species) for the removal of COD_Cr_, NH_4_^+^-N and TN (*p* < 0.05). The aeration at the bottom of the first quarter filtration chamber is just below the rhizosphere, which means that oxygen can be easily transmitted to it. However, TP were hardly affected by these two factors. This combined effect is consistent with the expectations that employing a suitable aeration position will further improve the COD_Cr_, NH_4_^+^-N and TN removal efficiencies in HSSF CWs used an optimized plant species.

## 4. Conclusions

The performances of six small scale HSSF-CW plots were investigated to determine the individual and combined effects of aeration position and plant species for water purification. Aeration at the bottom of the first quarter filtration chamber showed the highest removal efficiencies of COD_Cr_, NH_4_^+^-N and TN, which had better reoxygenation performance and benefited from the aerobic decomposition of organic matter and the nitrification process. CWs planted with *I. sibirica* exhibited significantly higher removal efficiencies of COD_C__r_ and NH_4_^+^-N than those planted with *C. indica* did (in both the warm and cool stages). And HSSF CWs planted with *I. sibirica* showed better TN removal efficiency than those planted with *C. indica* did in cool season. The interaction between aeration position and plant species had significant effects on the removal of COD_Cr_, NH_4_^+^-N and TN. Therefore, the plot aerated at the bottom of the first quarter filtration chamber and with *I. sibirica* remarkably outperformed the other HSSF CWs during the whole experiment period. The average TP removal efficiencies of the six HSSF-CW plots were not affected by either aeration position or plant species. Therefore, it is recommended that the combination of aeration at the bottom of the first quarter filtration chamber and the planting *I. sibirica* in the HSSF CWs could be a promising way for wastewater treatment, especially with regard to alleviating the low temperature negative effects on COD_Cr_ and NH_4_^+^-N removal in HSSF CWs during the cool season. Furthermore, the investigation of the enzyme activities and microbial community structures in the HSSF CWs, which could reveal the mechanism of combination measures to alleviate temperature changes, would be carried on.

## Figures and Tables

**Table 1:** F-values and significance of a two-way ANOVA for the effects of aeration position and plant species on HSSF CW treatment performance.

	Aeration Position	Plant Species	Aeration Position × Plant Species
COD_Cr_	19.5 ^a^	27.1 ^a^	3.31 ^b^
NH_4_^+^-N	9.86 ^a^	12.5 ^a^	3.49 ^b^
TN	8.74 ^a^	9.45 ^a^	5.35 ^a^
TP	2.29	1.68	0.27

^a^ *p* < 0.01, ^b^ *p* < 0.05.

## Data Availability

Data is contained within the article. The data presented in this study are available on https://doi.org/10.3390/ijerph19031583.
